# (η^6^-Isopropyl *N*-phenyl­carbamate)(η^5^-penta­methyl­cyclo­penta­dien­yl)ruthenium(II) tetra­phenyl­borate acetone monosolvate

**DOI:** 10.1107/S1600536811031655

**Published:** 2011-08-11

**Authors:** Bradley T. Loughrey, Michael L. Williams, Peter C. Healy

**Affiliations:** a Eskitis Institute for Cell and Molecular Therapies, Griffith University, Brisbane 4111, Australia; b School of Biomolecular and Physical Sciences, Griffith University, Brisbane 4111, Australia

## Abstract

The title complex, [Ru(C_10_H_15_)(C_10_H_13_NO_2_)](C_24_H_20_B)·C_3_H_6_O, is related to the analogous *O*-methyl complex. The average Ru—C distance to the penta­methyl­cyclo­penta­dienyl (Cp*) group is 2.19 (3) Å, and 2.21 (1) Å to the *ortho*, *meta* and *para* C atoms of the arene ring. The Ru—C_*ipso*_ bond length of 2.272 (3) Å is significantly longer, reflecting movement of the Ru atom away from the C atoms with electronegative substituents attached. The amide H atom in the cation forms an inter­molecular N—H⋯O hydrogen bond with the carbonyl O atom of the acetone solvent mol­ecule. A C—H⋯O inter­action also occurs.

## Related literature

For the synthesis and crystal structures of related compounds, see: Loughrey *et al.* (2008 ▶, 2009 ▶, 2010 ▶).
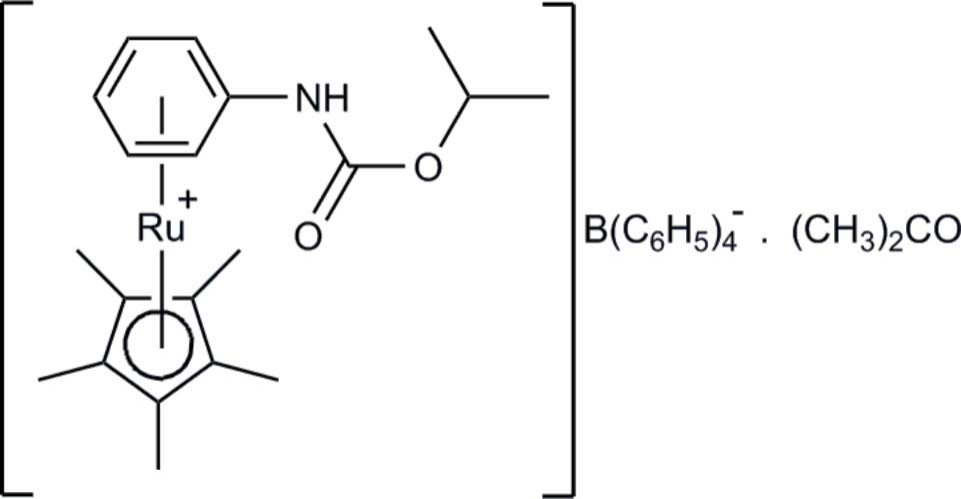


## Experimental

### 

#### Crystal data

[Ru(C_10_H_15_)(C_10_H_13_NO_2_)](C_24_H_20_B)·C_3_H_6_O
*M* *_r_* = 792.79
Monoclinic, 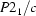

*a* = 9.8697 (2) Å
*b* = 28.7953 (7) Å
*c* = 14.3384 (3) Å
β = 92.334 (2)°
*V* = 4071.61 (15) Å^3^
*Z* = 4
Mo *K*α radiation
μ = 0.43 mm^−1^
*T* = 200 K
0.49 × 0.48 × 0.33 mm

#### Data collection

Oxford Diffraction Gemini S Ultra diffractometer
Absorption correction: multi-scan (*CrysAlis PRO*; Oxford Diffraction, 2010 ▶) *T*_min_ = 0.818, *T*_max_ = 0.872
44079 measured reflections
9342 independent reflections
8506 reflections with *I* > 2σ(*I*)
*R*_int_ = 0.025

#### Refinement

*R*[*F* ^2^ > 2σ(*F* ^2^)] = 0.048
*wR*(*F* ^2^) = 0.126
*S* = 1.15
9342 reflections
478 parameters
H-atom parameters constrained
Δρ_max_ = 1.18 e Å^−3^
Δρ_min_ = −1.20 e Å^−3^

### 

Data collection: *CrysAlis PRO* (Oxford Diffraction, 2010 ▶); cell refinement: *CrysAlis PRO*; data reduction: *CrysAlis PRO*; program(s) used to solve structure: *TEXSAN* (Molecular Structure Corporation, 2001 ▶) and *SIR97* (Altomare *et al.*, 1999 ▶); program(s) used to refine structure: *TEXSAN* and *SHELXL97* (Sheldrick, 2008 ▶); molecular graphics: *ORTEP-3 for Windows* (Farrugia, 1997 ▶); software used to prepare material for publication: *PLATON* (Spek, 2009 ▶) and *publCIF* (Westrip, 2010 ▶).

## Supplementary Material

Crystal structure: contains datablock(s) global, I. DOI: 10.1107/S1600536811031655/ng5208sup1.cif

Structure factors: contains datablock(s) I. DOI: 10.1107/S1600536811031655/ng5208Isup2.hkl

Additional supplementary materials:  crystallographic information; 3D view; checkCIF report

## Figures and Tables

**Table 1:** Hydrogen-bond geometry (Å, °)

*D*—H⋯*A*	*D*—H	H⋯*A*	*D*⋯*A*	*D*—H⋯*A*
N1—H1⋯O3	0.88	2.07	2.890 (4)	154
C6—H6*C*⋯O1	0.95	2.56	3.512 (5)	174

**Fig. 1.**
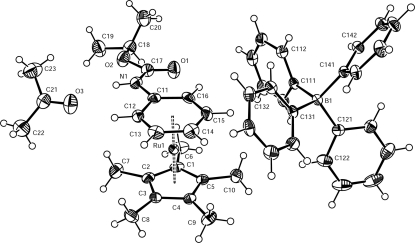
The structure of the cation and anion of (I), with atom labels and 40% probability displacement ellipsoids for the non-H atoms.

**Fig. 2.**
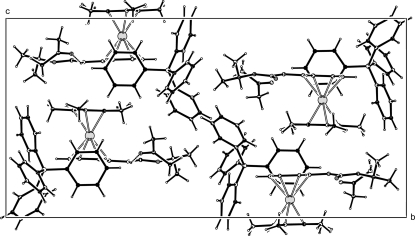
Crystal packing for (I), viewed down the a axis.

## supplementary crystallographic information

### Comment

As part of our ongoing investigations into the structural chemistry of full-sandwich Cp*Ru(II) η^6^-arene π-bonded complex salts [Cp*Ru(II)-arene]^+^*X*^-^ (Loughrey *et al.*, 2008, 2009, 2010), (Cp* = pentamethylcyclopentadiene) we have synthesized and determined the crystal structure of the title salt [Cp*Ru(η^6^-*O*-isopropyl *N*-phenylcarbamate)][BPh_4_] as its acetone solvate (Figure 1). The crystal lattice for this compound is isostructural with the *O*-methyl complex (Loughrey *et al.*, 2010) with cell dimensions for this latter complex *a* 9.8006 (8), *b* 27.610 (2), *c* 14.627 (1) Å, *β* 91.984 (7)°. The major difference in these parameters for the two compounds is the increase in the length of the *b* axis from 27.620 (2)Å to 28.7953 (7)Å in order to accomodate the increased steric bulk of the iso-propyl group.

In the cation, the average Ru—C distance to the Cp* group is 2.19 (3)Å and 2.21 (1)Å to the *ortho*, *meta* and *para* carbon atoms C12 - C16 of the arene group; in accord with results reported for other Cp*Ru(arene) sandwich complexes (Loughrey *et al.*, 2010 and references therein). The Ru—C*_ipso_* bond length of 2.272 (3)Å is, as observed for the other *O*-alkyl structures, significantly longer, reflecting movement of the Ru atom away from carbon atoms with electronegative substituents attached.

The carbamate group in the arene ligand adopts a *trans* conformation. The bond distances N1—C17 = 1.387 (5) Å, C17—O1 = 1.207 (5) Å, and C17—O2 = 1.339 (5) Å, are 0.02 - 0.03Å longer than the corresponding distances in the *O*-methyl, *O*-ethyl and *O*-propyl complexes (Loughrey *et al.*, 2010). The –NH—C(=O)—O—C– fragment is twisted out of the plane of the phenyl ring with the C17—O2—C11—C16 torsion angle 9.3 (5)°. The amide proton forms an intermolecular N—H···O hydrogen bond with the carbonyl oxygen of the acetone solvate.

The phenyl ring C11*n* (n = 1–6) of the [BPh_4_]^-^ anion and the arene ring are perpendicular to each other and form a head-to-tail polymeric array along the crystallographic *a* axis, with the *ortho* and *meta* protons of the arene ring 3.0Å above and below the plane of ring C11*n*. The methyl groups of the isopropyl substitutent and acetone solvate interact with the remaining three phenyl rings of the anion through C—H···π interactions (Figure 2).

### Experimental

Ruthenium trichloride hydrate (0.20 g,0.76 mmol) was transfered into a reaction vessel using iso-propanol (20 ml) and the solution heated at reflux until all starting material had dissolved. Phenylisocyanate (0.09 µ*L*, 0.76 mmol) and pentamethylcyclopentadiene (0.30 ml, 1.88 mmol) were added to the reaction mixture and the resulting solution heated under reflux conditions for a further 10 h. The solvent was concentrated under vacuum and the remaining residue dissolved in a water/diethyl ether partition mixture (20:20 ml). The aqueous portion was retained and washed with a further three portions of diethyl ether (20 ml). The aqueous layer was then mixed slowly with a solution of NaB(C_6_H_5_)_4(aq)_ (5 mL, 0.30 *M*) resulting in the formation of a white, crystalline precipitate. This material was filtered from solution, redissolved in a minimum quantity of acetone and filtered through a short alumina column (neutral, 150 mesh, acetone eluent). The eluent was concentrated under vacuum and the product recrystallized by allowing the acetone solvent to slowly evaporate. This process yielded crystals of the acetone solvate suitable for X-ray diffraction studies.

Yield = 0.241 g, 43.1%. ESMS (m/*z*): +ve ion, calcd m/*z* for [(η^5^-C_5_(CH_3_)_5_)Ru(η^6^-C_6_H_5_NHCO_2_CH(CH_3_)_2_)]^+^]: 415.56, found: 416.14 (100%), -ve ion,calcd m/*z* for B(C_6_H_5_)_4_^-^: 319.25,found: 319.18 (100%); NMR: ^1^H (d_6_DMSO), d 1.27 (d, 6H, CH(C*H*_3_)_2_),1.85 (s, 15H, C_5_(C_5_*H*_15_)), 3.33 (s,1*H*, *CH*(CH_3_)_2_),5.74–5.76 (m, 1H, aromatic), 5.87–5.90 (m, 2H, aromatic), 6.24–6.26 (m, 2H, aromatic),6.76–6.80 (m, 4H, B(C_6_H_5_)_4_*para*), 6.90–6.94 (m, 8H, B(C_6_H_5_)_4_*meta*), 7.17–7.19 (m, 8H, B(C_6_H_5_)_4_*ortho*), 9.78 (s, br, 1H, N*H*); ^13^C (d_6_DMSO),δ 9.81(C_5_(*C*_5_H_15_)), 21.66 (CH(*C*H_3_)_2_), 30.65 (*C*H(CH_3_)_2_), 77.25(aromatic), 85.24 (aromatic), 85.95 (aromatic), 95.13 (*C*_5_(C_5_H_15_), 110.01(*C-*NH), 121.49(4CH, B(C_6_H_5_)_4_^-^), 125.27 (8CH, B(C_6_H_5_)_4_^-^), 135.53 (8CH, B(C_6_H_5_)_4_)^-^, 153.21 (NH*C*O_2_), 162.62, 163.11, 163.60, 164.09 (4CH, B(C_6_H_5_)_4_), Signals Split by ^11^B).

### Refinement

H atoms attached to carbon and nitrogen were constrained as riding atoms, with C–H set to 0.94–96Å and N–H 0.88 Å. *U*_iso_(H) values were set to 1.2*U*_eq_ (N, aromatic) and 1.5*U*_eq_ (alkyl) of the parent atom.

### Crystal data

**Table:**

[Ru(C_10_H_15_)(C_10_H_13_NO_2_)](C_24_H_20_B)·C_3_H_6_O	*F*(000) = 1664
*M**_r_* = 792.79	*D*_x_ = 1.293 Mg m^−^^3^
Monoclinic, *P*2_1_/*c*	Mo *K*α radiation, λ = 0.71070 Å
Hall symbol: -P 2ybc	Cell parameters from 26954 reflections
*a* = 9.8697 (2) Å	θ = 3.2–32.4°
*b* = 28.7953 (7) Å	µ = 0.43 mm^−^^1^
*c* = 14.3384 (3) Å	*T* = 200 K
β = 92.334 (2)°	Block, colourless
*V* = 4071.61 (15) Å^3^	0.49 × 0.48 × 0.33 mm
*Z* = 4	

### Data collection

**Table:**

Oxford Diffraction Gemini S Ultra diffractometer	9342 independent reflections
Radiation source: Enhance (Mo) X-ray Source	8506 reflections with *I* > 2σ(*I*)
graphite	*R*_int_ = 0.025
Detector resolution: 16.0774 pixels mm^-1^	θ_max_ = 27.5°, θ_min_ = 3.3°
ω and φ scans	*h* = −12→12
Absorption correction: multi-scan (*CrysAlis PRO*; Oxford Diffraction, 2010)	*k* = −37→37
*T*_min_ = 0.818, *T*_max_ = 0.872	*l* = −18→18
44079 measured reflections	

### Refinement

**Table:**

Refinement on *F*^2^	Primary atom site location: structure-invariant direct methods
Least-squares matrix: full	Secondary atom site location: difference Fourier map
*R*[*F*^2^ > 2σ(*F*^2^)] = 0.048	Hydrogen site location: inferred from neighbouring sites
*wR*(*F*^2^) = 0.126	H-atom parameters constrained
*S* = 1.15	*w* = 1/[σ^2^(*F*_o_^2^) + (0.0522*P*)^2^ + 6.7463*P*] where *P* = (*F*_o_^2^ + 2*F*_c_^2^)/3
9342 reflections	(Δ/σ)_max_ < 0.001
478 parameters	Δρ_max_ = 1.18 e Å^−^^3^
0 restraints	Δρ_min_ = −1.20 e Å^−^^3^

### Special details

**Table:**

Experimental. CrysAlisPro, Oxford Diffraction Ltd. 2010, Version 1.171.34.14 (release 15-03-2010 CrysAlis171 .NET) Empirical absorption correction using spherical harmonics, implemented in SCALE3 ABSPACK scaling algorithm.
Geometry. Bond distances, angles *etc*. have been calculated using the rounded fractional coordinates. All su's are estimated from the variances of the (full) variance-covariance matrix. The cell e.s.d.'s are taken into account in the estimation of distances, angles and torsion angles
Refinement. Refinement on *F*^2^ for ALL reflections except those flagged by the user for potential systematic errors. Weighted *R*-factors *wR* and all goodnesses of fit *S* are based on *F*^2^, conventional *R*-factors *R* are based on *F*, with *F* set to zero for negative *F*^2^. The observed criterion of *F*^2^ > σ(*F*^2^) is used only for calculating -*R*-factor-obs *etc*. and is not relevant to the choice of reflections for refinement. *R*-factors based on *F*^2^ are statistically about twice as large as those based on *F*, and *R*-factors based on ALL data will be even larger.

### Fractional atomic coordinates and isotropic or equivalent isotropic displacement parameters (Å^2^)

**Table:**

	*x*	*y*	*z*	*U*_iso_*/*U*_eq_	
Ru1	0.44124 (2)	0.20924 (1)	0.41049 (1)	0.0230 (1)	
O1	0.2194 (3)	0.32596 (10)	0.3028 (2)	0.0573 (10)	
O2	0.3827 (3)	0.37946 (9)	0.2802 (2)	0.0518 (9)	
N1	0.4439 (3)	0.30608 (10)	0.2804 (2)	0.0365 (8)	
C1	0.3702 (3)	0.24487 (11)	0.53353 (19)	0.0333 (9)	
C2	0.5150 (4)	0.24541 (11)	0.5359 (2)	0.0364 (10)	
C3	0.5620 (3)	0.19837 (12)	0.5389 (2)	0.0326 (9)	
C4	0.4463 (3)	0.16854 (10)	0.5384 (2)	0.0300 (8)	
C5	0.3272 (3)	0.19730 (11)	0.5356 (2)	0.0297 (8)	
C6	0.2792 (5)	0.28640 (13)	0.5325 (3)	0.0523 (13)	
C7	0.6027 (5)	0.28816 (14)	0.5369 (3)	0.0576 (14)	
C8	0.7069 (4)	0.18270 (17)	0.5457 (3)	0.0525 (14)	
C9	0.4498 (4)	0.11682 (12)	0.5442 (3)	0.0463 (11)	
C10	0.1840 (4)	0.18072 (14)	0.5402 (3)	0.0447 (11)	
C11	0.4390 (3)	0.25776 (11)	0.28548 (19)	0.0302 (8)	
C12	0.5621 (3)	0.23262 (11)	0.2926 (2)	0.0318 (9)	
C13	0.5626 (3)	0.18379 (12)	0.2954 (2)	0.0379 (10)	
C14	0.4398 (4)	0.15916 (12)	0.2938 (2)	0.0431 (10)	
C15	0.3168 (4)	0.18361 (13)	0.2906 (2)	0.0397 (10)	
C16	0.3153 (3)	0.23274 (12)	0.2874 (2)	0.0344 (9)	
C17	0.3355 (4)	0.33617 (13)	0.2892 (2)	0.0422 (11)	
C18	0.2889 (4)	0.41772 (13)	0.2965 (3)	0.0483 (11)	
C19	0.3843 (4)	0.45778 (16)	0.3153 (4)	0.0649 (18)	
C20	0.1891 (4)	0.42725 (15)	0.2186 (3)	0.0573 (14)	
C111	−0.0628 (3)	0.15255 (9)	0.2436 (2)	0.0244 (7)	
C112	−0.0605 (3)	0.17988 (10)	0.1626 (2)	0.0307 (8)	
C113	−0.0551 (3)	0.22842 (11)	0.1645 (3)	0.0367 (9)	
C114	−0.0532 (3)	0.25171 (11)	0.2486 (3)	0.0405 (10)	
C115	−0.0581 (3)	0.22646 (12)	0.3296 (3)	0.0393 (10)	
C116	−0.0630 (3)	0.17789 (11)	0.3273 (2)	0.0311 (9)	
C121	−0.1220 (3)	0.07389 (10)	0.3350 (2)	0.0288 (8)	
C122	−0.0507 (4)	0.07272 (13)	0.4217 (2)	0.0415 (10)	
C123	−0.1053 (5)	0.05479 (15)	0.5020 (2)	0.0544 (15)	
C124	−0.2348 (5)	0.03813 (15)	0.4999 (3)	0.0580 (14)	
C125	−0.3100 (4)	0.03905 (14)	0.4169 (3)	0.0501 (12)	
C126	−0.2542 (3)	0.05708 (11)	0.3365 (2)	0.0358 (9)	
C131	0.1017 (3)	0.07966 (9)	0.2258 (2)	0.0250 (7)	
C132	0.1706 (3)	0.09768 (10)	0.1506 (2)	0.0303 (8)	
C133	0.3009 (3)	0.08441 (11)	0.1300 (2)	0.0338 (9)	
C134	0.3705 (3)	0.05224 (11)	0.1852 (2)	0.0356 (10)	
C135	0.3067 (3)	0.03349 (12)	0.2595 (3)	0.0417 (10)	
C136	0.1742 (3)	0.04664 (11)	0.2788 (2)	0.0370 (9)	
C141	−0.1450 (3)	0.07536 (9)	0.14778 (19)	0.0242 (7)	
C142	−0.2656 (3)	0.09578 (11)	0.1127 (2)	0.0298 (8)	
C143	−0.3409 (3)	0.07764 (13)	0.0372 (2)	0.0369 (10)	
C144	−0.2990 (3)	0.03742 (13)	−0.0059 (2)	0.0407 (10)	
C145	−0.1806 (3)	0.01606 (12)	0.0265 (2)	0.0405 (10)	
C146	−0.1062 (3)	0.03479 (10)	0.1019 (2)	0.0311 (8)	
B1	−0.0576 (3)	0.09523 (11)	0.2396 (2)	0.0233 (8)	
O3	0.7186 (3)	0.33650 (10)	0.3225 (3)	0.0645 (10)	
C21	0.7980 (4)	0.36894 (13)	0.3270 (3)	0.0433 (11)	
C22	0.9161 (4)	0.36791 (18)	0.3934 (3)	0.0608 (14)	
C23	0.7796 (6)	0.40992 (17)	0.2656 (4)	0.0714 (19)	
H1	0.53090	0.31230	0.27500	0.0280*	
H6A	0.19310	0.27790	0.55350	0.0640*	
H6B	0.31820	0.30970	0.57210	0.0640*	
H6C	0.26980	0.29820	0.47040	0.0640*	
H7A	0.68020	0.28310	0.57730	0.0690*	
H7B	0.63100	0.29420	0.47560	0.0690*	
H7C	0.55280	0.31380	0.55870	0.0690*	
H8A	0.71170	0.15190	0.56920	0.0630*	
H8B	0.74360	0.18330	0.48490	0.0630*	
H8C	0.75790	0.20290	0.58560	0.0630*	
H9A	0.36490	0.10570	0.56510	0.0560*	
H9B	0.46400	0.10430	0.48390	0.0560*	
H9C	0.52030	0.10730	0.58630	0.0560*	
H10A	0.13660	0.19980	0.58200	0.0530*	
H10B	0.14050	0.18270	0.47960	0.0530*	
H10C	0.18300	0.14950	0.56090	0.0530*	
H12	0.64580	0.24900	0.29530	0.0390*	
H13	0.64610	0.16760	0.29830	0.0460*	
H14	0.43980	0.12630	0.29470	0.0500*	
H15	0.23290	0.16710	0.29050	0.0490*	
H16	0.23130	0.24880	0.28630	0.0400*	
H18	0.24200	0.41170	0.35150	0.0580*	
H19A	0.46910	0.44700	0.34130	0.0770*	
H19B	0.40060	0.47410	0.25860	0.0770*	
H19C	0.34650	0.47910	0.35820	0.0770*	
H20A	0.21530	0.45410	0.18400	0.0670*	
H20B	0.18360	0.40140	0.17660	0.0670*	
H20C	0.10130	0.43270	0.24180	0.0670*	
H112	−0.06270	0.16490	0.10350	0.0380*	
H113	−0.05160	0.24510	0.10810	0.0440*	
H114	−0.04920	0.28470	0.24960	0.0480*	
H115	−0.05800	0.24210	0.38780	0.0480*	
H116	−0.06720	0.16150	0.38430	0.0370*	
H122	0.03880	0.08480	0.42580	0.0500*	
H123	−0.05280	0.05450	0.55910	0.0660*	
H124	−0.27330	0.02550	0.55430	0.0700*	
H125	−0.40140	0.02810	0.41420	0.0610*	
H126	−0.30830	0.05780	0.28030	0.0440*	
H132	0.12600	0.11980	0.11150	0.0350*	
H133	0.34330	0.09780	0.07750	0.0410*	
H134	0.46040	0.04310	0.17160	0.0430*	
H135	0.35220	0.01150	0.29800	0.0500*	
H136	0.13270	0.03280	0.33070	0.0440*	
H142	−0.29730	0.12310	0.14140	0.0350*	
H143	−0.42230	0.09280	0.01560	0.0440*	
H144	−0.35010	0.02510	−0.05790	0.0500*	
H145	−0.15080	−0.01170	−0.00240	0.0480*	
H146	−0.02530	0.01950	0.12270	0.0390*	
H22A	0.88880	0.35970	0.45460	0.0700*	
H22B	0.98220	0.34600	0.37460	0.0700*	
H22C	0.95870	0.39800	0.39740	0.0700*	
H23A	0.80170	0.43770	0.29910	0.0870*	
H23B	0.83740	0.40770	0.21360	0.0870*	
H23C	0.68790	0.41180	0.24210	0.0870*	

### Atomic displacement parameters (Å^2^)

**Table:**

	*U*^11^	*U*^22^	*U*^33^	*U*^12^	*U*^13^	*U*^23^
Ru1	0.0275 (1)	0.0226 (1)	0.0189 (1)	−0.0033 (1)	0.0001 (1)	−0.0046 (1)
O1	0.0377 (14)	0.0517 (16)	0.083 (2)	−0.0029 (12)	0.0079 (13)	0.0006 (15)
O2	0.0437 (14)	0.0396 (14)	0.0727 (19)	0.0004 (11)	0.0098 (13)	0.0059 (13)
N1	0.0370 (14)	0.0351 (14)	0.0374 (15)	−0.0007 (11)	0.0025 (11)	0.0053 (11)
C1	0.0537 (19)	0.0306 (15)	0.0156 (13)	0.0042 (13)	0.0023 (12)	−0.0045 (11)
C2	0.056 (2)	0.0370 (16)	0.0157 (13)	−0.0142 (14)	−0.0029 (12)	−0.0006 (11)
C3	0.0344 (16)	0.0433 (17)	0.0198 (14)	−0.0054 (13)	−0.0018 (11)	0.0024 (12)
C4	0.0354 (15)	0.0325 (15)	0.0224 (13)	0.0009 (12)	0.0039 (11)	0.0004 (11)
C5	0.0337 (15)	0.0339 (15)	0.0218 (13)	0.0018 (12)	0.0035 (11)	0.0007 (11)
C6	0.085 (3)	0.0369 (19)	0.0353 (19)	0.0181 (18)	0.0075 (19)	−0.0045 (14)
C7	0.083 (3)	0.051 (2)	0.038 (2)	−0.034 (2)	−0.0071 (19)	−0.0068 (16)
C8	0.0368 (19)	0.079 (3)	0.041 (2)	−0.0022 (18)	−0.0061 (15)	0.0107 (19)
C9	0.056 (2)	0.0317 (17)	0.052 (2)	0.0030 (15)	0.0106 (17)	0.0082 (15)
C10	0.0352 (17)	0.057 (2)	0.0425 (19)	−0.0028 (15)	0.0084 (14)	0.0041 (16)
C11	0.0414 (16)	0.0333 (15)	0.0157 (12)	−0.0005 (12)	−0.0002 (11)	−0.0025 (11)
C12	0.0299 (14)	0.0438 (17)	0.0221 (13)	−0.0040 (12)	0.0061 (11)	−0.0049 (12)
C13	0.0440 (18)	0.0445 (18)	0.0255 (15)	0.0113 (14)	0.0053 (13)	−0.0113 (13)
C14	0.067 (2)	0.0320 (16)	0.0302 (16)	−0.0056 (15)	0.0013 (15)	−0.0147 (13)
C15	0.0436 (18)	0.0483 (19)	0.0269 (15)	−0.0179 (15)	−0.0023 (13)	−0.0123 (14)
C16	0.0317 (15)	0.0503 (19)	0.0208 (14)	0.0010 (13)	−0.0037 (11)	−0.0058 (13)
C17	0.0410 (18)	0.0451 (19)	0.0405 (19)	−0.0046 (15)	0.0002 (14)	0.0013 (15)
C18	0.0427 (19)	0.0413 (19)	0.062 (2)	0.0075 (15)	0.0159 (17)	0.0035 (17)
C19	0.047 (2)	0.056 (3)	0.091 (4)	−0.0011 (19)	−0.006 (2)	0.002 (2)
C20	0.055 (2)	0.052 (2)	0.065 (3)	−0.0038 (18)	0.003 (2)	−0.007 (2)
C111	0.0164 (11)	0.0264 (13)	0.0304 (14)	0.0021 (9)	−0.0002 (10)	−0.0038 (11)
C112	0.0279 (14)	0.0305 (14)	0.0339 (15)	0.0020 (11)	0.0023 (11)	−0.0019 (12)
C113	0.0305 (15)	0.0285 (15)	0.0510 (19)	0.0019 (12)	0.0002 (13)	0.0070 (13)
C114	0.0281 (15)	0.0242 (14)	0.069 (2)	0.0029 (11)	0.0009 (15)	−0.0068 (15)
C115	0.0344 (16)	0.0344 (16)	0.049 (2)	0.0020 (13)	0.0012 (14)	−0.0168 (15)
C116	0.0260 (14)	0.0324 (15)	0.0348 (16)	0.0005 (11)	0.0010 (11)	−0.0066 (12)
C121	0.0357 (15)	0.0238 (13)	0.0268 (14)	0.0037 (11)	0.0011 (11)	−0.0030 (11)
C122	0.0460 (19)	0.0482 (19)	0.0300 (16)	0.0094 (15)	−0.0003 (14)	−0.0013 (14)
C123	0.071 (3)	0.065 (3)	0.0272 (17)	0.023 (2)	0.0030 (16)	0.0068 (16)
C124	0.084 (3)	0.056 (2)	0.036 (2)	0.017 (2)	0.026 (2)	0.0142 (17)
C125	0.054 (2)	0.047 (2)	0.051 (2)	−0.0047 (17)	0.0241 (18)	0.0030 (17)
C126	0.0411 (17)	0.0347 (16)	0.0320 (16)	−0.0027 (13)	0.0084 (13)	−0.0018 (12)
C131	0.0223 (12)	0.0228 (12)	0.0297 (14)	−0.0014 (10)	−0.0013 (10)	−0.0065 (10)
C132	0.0277 (14)	0.0293 (14)	0.0337 (15)	0.0014 (11)	−0.0016 (11)	−0.0036 (12)
C133	0.0281 (14)	0.0338 (15)	0.0400 (17)	−0.0026 (12)	0.0060 (12)	−0.0073 (13)
C134	0.0228 (14)	0.0301 (15)	0.054 (2)	0.0005 (11)	0.0023 (13)	−0.0111 (14)
C135	0.0330 (16)	0.0368 (17)	0.055 (2)	0.0111 (13)	−0.0009 (14)	0.0040 (15)
C136	0.0309 (15)	0.0355 (16)	0.0446 (18)	0.0046 (12)	0.0029 (13)	0.0061 (14)
C141	0.0213 (12)	0.0273 (13)	0.0241 (13)	−0.0026 (10)	0.0040 (10)	−0.0025 (10)
C142	0.0251 (13)	0.0357 (15)	0.0285 (14)	0.0013 (11)	0.0017 (11)	−0.0036 (12)
C143	0.0251 (14)	0.0517 (19)	0.0336 (16)	−0.0005 (13)	−0.0018 (12)	−0.0033 (14)
C144	0.0366 (17)	0.055 (2)	0.0302 (16)	−0.0106 (15)	−0.0033 (13)	−0.0133 (15)
C145	0.0388 (17)	0.0419 (18)	0.0411 (18)	−0.0041 (14)	0.0055 (14)	−0.0192 (15)
C146	0.0250 (13)	0.0317 (15)	0.0368 (16)	−0.0017 (11)	0.0031 (11)	−0.0086 (12)
B1	0.0215 (14)	0.0245 (14)	0.0237 (14)	0.0003 (11)	0.0001 (11)	−0.0023 (11)
O3	0.0510 (17)	0.0503 (16)	0.093 (2)	−0.0068 (13)	0.0117 (16)	−0.0071 (16)
C21	0.0417 (18)	0.0432 (19)	0.046 (2)	0.0065 (15)	0.0156 (15)	−0.0058 (15)
C22	0.054 (2)	0.079 (3)	0.050 (2)	0.008 (2)	0.0092 (18)	−0.011 (2)
C23	0.094 (4)	0.059 (3)	0.061 (3)	−0.003 (3)	0.002 (3)	0.010 (2)

### Geometric parameters (Å, °)

**Table:**

Ru1—C1	2.181 (3)	C20—H20A	0.9600
Ru1—C2	2.178 (3)	C20—H20B	0.9600
Ru1—C3	2.175 (3)	C20—H20C	0.9500
Ru1—C4	2.175 (3)	C111—C112	1.404 (4)
Ru1—C5	2.184 (3)	C111—C116	1.405 (4)
Ru1—C11	2.272 (3)	C111—B1	1.652 (4)
Ru1—C12	2.213 (3)	C112—C113	1.399 (4)
Ru1—C13	2.204 (3)	C113—C114	1.379 (6)
Ru1—C14	2.208 (3)	C114—C115	1.373 (6)
Ru1—C15	2.199 (3)	C115—C116	1.400 (5)
Ru1—C16	2.222 (3)	C121—C126	1.393 (4)
O1—C17	1.207 (5)	C121—B1	1.650 (4)
O2—C17	1.339 (5)	C121—C122	1.404 (4)
O2—C18	1.464 (5)	C122—C123	1.390 (5)
O3—C21	1.219 (5)	C123—C124	1.364 (7)
N1—C11	1.394 (4)	C124—C125	1.377 (6)
N1—C17	1.387 (5)	C125—C126	1.398 (5)
N1—H1	0.8800	C131—C136	1.396 (4)
C1—C5	1.435 (4)	C131—B1	1.655 (4)
C1—C6	1.495 (5)	C131—C132	1.398 (4)
C1—C2	1.428 (5)	C132—C133	1.385 (4)
C2—C7	1.505 (6)	C133—C134	1.383 (4)
C2—C3	1.432 (5)	C134—C135	1.370 (5)
C3—C4	1.429 (4)	C135—C136	1.400 (4)
C3—C8	1.499 (5)	C141—C142	1.402 (4)
C4—C5	1.437 (4)	C141—C146	1.402 (4)
C4—C9	1.492 (5)	C141—B1	1.647 (4)
C5—C10	1.496 (5)	C142—C143	1.390 (4)
C11—C16	1.419 (4)	C143—C144	1.384 (5)
C11—C12	1.414 (4)	C144—C145	1.384 (4)
C12—C13	1.407 (5)	C145—C146	1.391 (4)
C13—C14	1.404 (5)	C112—H112	0.9500
C14—C15	1.403 (5)	C113—H113	0.9400
C15—C16	1.416 (5)	C114—H114	0.9500
C18—C20	1.484 (6)	C115—H115	0.9500
C18—C19	1.506 (6)	C116—H116	0.9500
C6—H6A	0.9500	C21—C22	1.475 (6)
C6—H6B	0.9500	C21—C23	1.479 (7)
C6—H6C	0.9500	C122—H122	0.9500
C7—H7A	0.9500	C123—H123	0.9500
C7—H7B	0.9500	C124—H124	0.9500
C7—H7C	0.9500	C125—H125	0.9500
C8—H8B	0.9600	C126—H126	0.9500
C8—H8C	0.9500	C132—H132	0.9500
C8—H8A	0.9500	C133—H133	0.9600
C9—H9B	0.9500	C134—H134	0.9500
C9—H9C	0.9400	C135—H135	0.9400
C9—H9A	0.9600	C136—H136	0.9500
C10—H10B	0.9600	C142—H142	0.9500
C10—H10C	0.9500	C143—H143	0.9500
C10—H10A	0.9500	C144—H144	0.9500
C12—H12	0.9500	C145—H145	0.9500
C13—H13	0.9500	C146—H146	0.9500
C14—H14	0.9500	C22—H22A	0.9600
C15—H15	0.9500	C22—H22B	0.9500
C16—H16	0.9500	C22—H22C	0.9600
C18—H18	0.9500	C23—H23A	0.9500
C19—H19A	0.9500	C23—H23B	0.9600
C19—H19C	0.9600	C23—H23C	0.9500
C19—H19B	0.9600		
			
O1···C16	2.858 (4)	C133···H14	2.9400
O1···C20	3.166 (5)	C134···H144^ix^	2.8800
O3···C12	3.386 (4)	C134···H14	2.7200
O3···N1	2.890 (4)	C135···H23A^x^	3.0600
O1···H16	2.2400	C135···H14	3.0100
O1···H18	2.5700	C136···H122	2.7700
O1···H22B^i^	2.6600	C136···H146	3.0200
O1···H20B	2.8400	C141···H126	2.5900
O1···H6C	2.5600	C141···H132	3.0300
O3···H1	2.0700	C141···H112	2.7800
O3···H7B	2.6800	C142···H112	2.8300
O3···H12	2.6400	C142···H22A^viii^	3.0600
N1···O3	2.890 (4)	C142···H126	2.6900
C1···C16	3.566 (4)	C143···H134^i^	2.9800
C1···C4	2.323 (4)	C144···H23A^viii^	3.0900
C1···C7	2.610 (6)	C144···H19C^vii^	2.7600
C1···C3	2.317 (4)	C145···H19C^vii^	2.6000
C1···C10	2.610 (5)	C146···H19C^vii^	2.9400
C2···C5	2.314 (5)	C146···H145^ix^	3.0300
C2···C6	2.608 (6)	H1···H12	2.1600
C2···C4	2.316 (4)	H1···O3	2.0700
C2···C12	3.557 (4)	H6A···C10	2.8100
C2···C8	2.617 (6)	H6A···C113^iii^	2.9800
C3···C13	3.517 (4)	H6A···H10A	2.3600
C3···C9	2.599 (5)	H6B···H7C	2.3300
C3···C1	2.317 (4)	H6B···C7	2.9400
C3···C5	2.316 (4)	H6C···C17	2.9200
C3···C7	2.617 (5)	H6C···O1	2.5600
C4···C14	3.516 (4)	H7A···C8	2.9400
C4···C10	2.614 (5)	H7A···C113^xi^	2.8700
C4···C8	2.602 (5)	H7A···H8C	2.4300
C4···C1	2.323 (4)	H7A···C112^xi^	2.9900
C4···C2	2.316 (4)	H7B···O3	2.6800
C5···C9	2.615 (5)	H7C···H6B	2.3300
C5···C6	2.609 (5)	H7C···C6	2.8200
C5···C15	3.532 (4)	H7C···H142^xi^	2.6000
C5···C2	2.314 (5)	H8A···H9C	2.3100
C5···C3	2.316 (4)	H8A···C9	2.7800
C11···C14	2.842 (5)	H8B···H116^vi^	2.4900
C11···C15	2.455 (5)	H8B···C116^vi^	3.0200
C11···C13	2.456 (5)	H8C···C7	2.9600
C12···O3	3.386 (4)	H8C···H7A	2.4300
C12···C14	2.436 (5)	H8C···C113^xi^	2.9000
C12···C2	3.557 (4)	H8C···H113^xi^	2.4100
C12···C16	2.434 (4)	H9A···H20B^iii^	2.4600
C12···N1	2.419 (4)	H9A···C20^iii^	3.0100
C12···C15	2.802 (5)	H9A···C10	2.8200
C13···C3	3.517 (4)	H9A···H10C	2.1900
C13···C11	2.456 (5)	H9B···C125^vi^	3.1000
C13···C16	2.817 (4)	H9C···H8A	2.3100
C13···C15	2.424 (5)	H9C···C8	2.9200
C14···C12	2.436 (5)	H10A···H6A	2.3600
C14···C16	2.449 (5)	H10A···H113^iii^	2.4800
C14···C133	3.430 (4)	H10A···C6	2.9600
C14···C11	2.842 (5)	H10A···C113^iii^	3.0700
C14···C134	3.505 (5)	H10B···H116	2.4900
C14···C4	3.516 (4)	H10B···C116	2.9100
C15···C12	2.802 (5)	H10C···C9	2.8200
C15···C11	2.455 (5)	H10C···H20B^iii^	2.2100
C15···C132	3.464 (5)	H10C···H9A	2.1900
C15···C13	2.424 (5)	H12···C114^vi^	3.0700
C15···C5	3.532 (4)	H12···C115^vi^	3.0100
C16···N1	2.468 (4)	H12···O3	2.6400
C16···C1	3.566 (4)	H12···H1	2.1600
C16···O1	2.858 (4)	H13···C116^vi^	2.9000
C16···C12	2.434 (4)	H13···C111^vi^	3.0400
C16···C14	2.449 (5)	H14···C135	3.0100
C16···C13	2.817 (4)	H14···C134	2.7200
C19···C145^ii^	3.518 (6)	H14···C133	2.9400
C20···O1	3.166 (5)	H15···C116	3.0000
C6···H7C	2.8200	H15···C131	2.9600
C6···H10A	2.9600	H15···C132	2.8800
C7···H8C	2.9600	H15···C111	3.0000
C7···H6B	2.9400	H16···O1	2.2400
C8···H7A	2.9400	H16···C17	2.7200
C8···H9C	2.9200	H16···C114	2.8400
C9···H10C	2.8200	H16···C115	3.0200
C9···H8A	2.7800	H18···O1	2.5700
C10···H20B^iii^	3.0700	H19A···H144^xi^	2.3900
C10···H9A	2.8200	H19B···H134^iv^	2.5900
C10···H6A	2.8100	H19B···C126^ii^	3.0800
C11···H16	2.0700	H19B···H20A	2.1600
C11···H12	2.0600	H19C···C145^ii^	2.6000
C12···H13	2.0500	H19C···C144^ii^	2.7600
C112···C132	3.297 (4)	H19C···C146^ii^	2.9400
C112···C142	3.218 (4)	H20A···C126^ii^	3.0100
C13···H12	2.0500	H20A···H19B	2.1600
C13···H14	2.0500	H20A···C125^ii^	3.0100
C14···H15	2.0500	H20B···C17	2.8600
C14···H13	2.0500	H20B···H10C^v^	2.2100
C15···H14	2.0500	H20B···C10^v^	3.0700
C15···H16	2.0600	H20B···O1	2.8400
C116···C122	3.317 (5)	H20B···H9A^v^	2.4600
C16···H15	2.0600	H22A···C142^xi^	3.0600
C17···H6C	2.9200	H22A···H112^xi^	2.2800
C17···H16	2.7200	H22B···H114^vi^	2.5300
C17···H20B	2.8600	H22B···O1^vi^	2.6600
C19···H134^iv^	2.9000	H22C···H23A	2.3500
C20···H9A^v^	3.0100	H23A···H22C	2.3500
C21···H114^vi^	3.0900	H23A···C144^xi^	3.0900
C122···C136	3.172 (5)	H23A···C135^iv^	3.0600
C122···C116	3.317 (5)	H112···C141	2.7800
C126···C142	3.395 (4)	H112···C132	3.0600
C132···C15	3.464 (5)	H112···H22A^viii^	2.2800
C132···C112	3.297 (4)	H112···C142	2.8300
C132···C146	3.328 (4)	H112···H132	2.2700
C133···C14	3.430 (4)	H113···H8C^viii^	2.4100
C134···C14	3.505 (5)	H113···H10A^v^	2.4800
C136···C122	3.172 (5)	H114···H22B^i^	2.5300
C142···C126	3.395 (4)	H114···C21^i^	3.0900
C142···C112	3.218 (4)	H116···H122	2.5100
C145···C19^vii^	3.518 (6)	H116···C121	2.6700
C146···C132	3.328 (4)	H116···C122	2.6200
C111···H142	2.8200	H116···H8B^i^	2.4900
C111···H132	2.8700	H116···H10B	2.4900
C111···H13^i^	3.0400	H122···C136	2.7700
C111···H15	3.0000	H122···H116	2.5100
C112···H7A^viii^	2.9900	H122···C131	2.9600
C112···H142	2.8600	H122···H136	2.2500
C112···H132	2.6500	H124···H135^xii^	2.5200
C113···H10A^v^	3.0700	H126···C142	2.6900
C113···H7A^viii^	2.8700	H126···C141	2.5900
C113···H8C^viii^	2.9000	H132···C112	2.6500
C113···H6A^v^	2.9800	H132···C141	3.0300
C114···H12^i^	3.0700	H132···C111	2.8700
C114···H16	2.8400	H132···H112	2.2700
C115···H12^i^	3.0100	H133···H143^vi^	2.5200
C115···H16	3.0200	H134···C19^x^	2.9000
C116···H10B	2.9100	H134···H19B^x^	2.5900
C116···H15	3.0000	H134···C143^vi^	2.9800
C116···H13^i^	2.9000	H135···H124^xii^	2.5200
C116···H8B^i^	3.0200	H136···H122	2.2500
C121···H136	2.7800	H136···C121	2.7800
C121···H116	2.6700	H136···C122	2.5500
C122···H136	2.5500	H142···H7C^viii^	2.6000
C122···H116	2.6200	H142···C111	2.8200
C125···H20A^vii^	3.0100	H142···C112	2.8600
C125···H9B^i^	3.1000	H143···H133^i^	2.5200
C126···H19B^vii^	3.0800	H144···C134^ix^	2.8800
C126···H20A^vii^	3.0100	H144···H19A^viii^	2.3900
C131···H122	2.9600	H145···C146^ix^	3.0300
C131···H146	2.5700	H145···H146^ix^	2.5100
C131···H15	2.9600	H146···H145^ix^	2.5100
C132···H146	2.9800	H146···C131	2.5700
C132···H15	2.8800	H146···C132	2.9800
C132···H112	3.0600	H146···C136	3.0200
			
C1—Ru1—C2	38.26 (13)	C4—C9—H9B	109.00
C1—Ru1—C3	64.28 (11)	C4—C9—H9C	110.00
C1—Ru1—C4	64.44 (11)	H9A—C9—H9B	109.00
C1—Ru1—C5	38.38 (12)	H9A—C9—H9C	110.00
C1—Ru1—C11	110.84 (11)	H9B—C9—H9C	110.00
C1—Ru1—C12	132.51 (11)	C5—C10—H10A	109.00
C1—Ru1—C13	164.71 (11)	C5—C10—H10B	109.00
C1—Ru1—C14	158.06 (13)	C5—C10—H10C	110.00
C1—Ru1—C15	127.04 (12)	H10A—C10—H10B	109.00
C1—Ru1—C16	108.14 (11)	H10A—C10—H10C	110.00
C2—Ru1—C3	38.41 (13)	H10B—C10—H10C	109.00
C2—Ru1—C4	64.28 (11)	Ru1—C12—H12	128.00
C2—Ru1—C5	64.09 (12)	C11—C12—H12	119.00
C2—Ru1—C11	110.46 (11)	C13—C12—H12	120.00
C2—Ru1—C12	108.23 (12)	Ru1—C13—H13	129.00
C2—Ru1—C13	127.15 (13)	C12—C13—H13	120.00
C2—Ru1—C14	158.37 (14)	C14—C13—H13	120.00
C2—Ru1—C15	164.43 (14)	Ru1—C14—H14	130.00
C2—Ru1—C16	131.88 (12)	C13—C14—H14	120.00
C3—Ru1—C4	38.35 (11)	C15—C14—H14	120.00
C3—Ru1—C5	64.20 (11)	Ru1—C15—H15	129.00
C3—Ru1—C11	138.06 (11)	C14—C15—H15	120.00
C3—Ru1—C12	113.36 (11)	C16—C15—H15	119.00
C3—Ru1—C13	106.87 (11)	Ru1—C16—H16	128.00
C3—Ru1—C14	122.26 (13)	C11—C16—H16	120.00
C3—Ru1—C15	151.99 (13)	C15—C16—H16	120.00
C3—Ru1—C16	170.28 (13)	O2—C18—H18	109.00
C4—Ru1—C5	38.50 (11)	C19—C18—H18	108.00
C4—Ru1—C11	174.61 (11)	C20—C18—H18	109.00
C4—Ru1—C12	144.42 (11)	C18—C19—H19A	111.00
C4—Ru1—C13	117.24 (11)	C18—C19—H19B	110.00
C4—Ru1—C14	106.63 (11)	C18—C19—H19C	110.00
C4—Ru1—C15	118.11 (12)	H19A—C19—H19B	108.00
C4—Ru1—C16	145.74 (11)	H19A—C19—H19C	108.00
C5—Ru1—C11	139.13 (11)	H19B—C19—H19C	108.00
C5—Ru1—C12	170.88 (12)	C18—C20—H20A	111.00
C5—Ru1—C13	151.28 (12)	C18—C20—H20B	110.00
C5—Ru1—C14	122.14 (12)	C18—C20—H20C	111.00
C5—Ru1—C15	107.41 (12)	H20A—C20—H20B	108.00
C5—Ru1—C16	114.21 (11)	H20A—C20—H20C	108.00
C11—Ru1—C12	36.74 (11)	H20B—C20—H20C	109.00
C11—Ru1—C13	66.54 (11)	C112—C111—C116	114.6 (2)
C11—Ru1—C14	78.72 (11)	C112—C111—B1	122.0 (2)
C11—Ru1—C15	66.58 (12)	C116—C111—B1	123.4 (2)
C11—Ru1—C16	36.79 (11)	C111—C112—C113	123.1 (3)
C12—Ru1—C13	37.14 (12)	C112—C113—C114	120.1 (3)
C12—Ru1—C14	66.86 (12)	C113—C114—C115	118.9 (3)
C12—Ru1—C15	78.83 (12)	C114—C115—C116	120.8 (4)
C12—Ru1—C16	66.56 (11)	C111—C116—C115	122.5 (3)
C13—Ru1—C14	37.10 (13)	C122—C121—C126	114.5 (3)
C13—Ru1—C15	66.82 (12)	C122—C121—B1	123.2 (3)
C13—Ru1—C16	79.05 (11)	C126—C121—B1	122.3 (2)
C14—Ru1—C15	37.11 (14)	C121—C122—C123	123.0 (4)
C14—Ru1—C16	67.11 (12)	C122—C123—C124	120.4 (3)
C15—Ru1—C16	37.34 (13)	C123—C124—C125	118.9 (4)
C17—O2—C18	117.4 (3)	C124—C125—C126	120.2 (4)
C11—N1—C17	126.2 (3)	C121—C126—C125	122.9 (3)
C11—N1—H1	104.00	C132—C131—C136	114.7 (3)
C17—N1—H1	130.00	C132—C131—B1	119.1 (2)
Ru1—C1—C2	70.74 (17)	C136—C131—B1	126.0 (2)
C2—C1—C5	107.9 (3)	C131—C132—C133	123.2 (3)
Ru1—C1—C5	70.90 (17)	C132—C133—C134	120.5 (3)
Ru1—C1—C6	125.5 (2)	C133—C134—C135	118.4 (3)
C5—C1—C6	125.9 (3)	C134—C135—C136	120.6 (3)
C2—C1—C6	126.3 (3)	C131—C136—C135	122.6 (3)
Ru1—C2—C3	70.69 (17)	C142—C141—C146	115.2 (3)
Ru1—C2—C1	71.01 (18)	C142—C141—B1	123.4 (2)
C1—C2—C7	125.7 (3)	C146—C141—B1	121.4 (2)
Ru1—C2—C7	124.9 (2)	C141—C142—C143	122.8 (3)
C1—C2—C3	108.2 (3)	C142—C143—C144	120.2 (3)
C3—C2—C7	126.0 (3)	C143—C144—C145	119.1 (3)
Ru1—C3—C8	125.9 (2)	C144—C145—C146	120.0 (3)
C2—C3—C4	108.1 (3)	C141—C146—C145	122.8 (3)
Ru1—C3—C4	70.85 (16)	C113—C112—H112	118.00
C4—C3—C8	125.4 (3)	C111—C112—H112	119.00
C2—C3—C8	126.4 (3)	C112—C113—H113	120.00
Ru1—C3—C2	70.90 (17)	C114—C113—H113	120.00
Ru1—C4—C3	70.81 (17)	C115—C114—H114	121.00
C3—C4—C5	107.8 (3)	C113—C114—H114	120.00
C3—C4—C9	125.7 (3)	C114—C115—H115	120.00
C5—C4—C9	126.4 (3)	C116—C115—H115	120.00
Ru1—C4—C9	125.8 (2)	C111—C116—H116	119.00
Ru1—C4—C5	71.05 (16)	C115—C116—H116	119.00
Ru1—C5—C4	70.45 (16)	C22—C21—C23	118.4 (4)
Ru1—C5—C1	70.72 (16)	O3—C21—C23	121.1 (4)
C1—C5—C10	125.9 (3)	O3—C21—C22	120.5 (4)
C4—C5—C10	126.0 (3)	C121—C122—H122	119.00
C1—C5—C4	108.0 (3)	C123—C122—H122	118.00
Ru1—C5—C10	127.3 (2)	C122—C123—H123	120.00
Ru1—C11—C12	69.37 (17)	C124—C123—H123	120.00
Ru1—C11—N1	131.0 (2)	C123—C124—H124	121.00
N1—C11—C16	122.7 (3)	C125—C124—H124	120.00
Ru1—C11—C16	69.71 (17)	C126—C125—H125	120.00
N1—C11—C12	118.9 (3)	C124—C125—H125	120.00
C12—C11—C16	118.4 (3)	C125—C126—H126	119.00
Ru1—C12—C13	71.06 (17)	C121—C126—H126	118.00
C11—C12—C13	121.0 (3)	C131—C132—H132	119.00
Ru1—C12—C11	73.90 (17)	C133—C132—H132	118.00
C12—C13—C14	120.2 (3)	C134—C133—H133	120.00
Ru1—C13—C12	71.79 (17)	C132—C133—H133	120.00
Ru1—C13—C14	71.64 (18)	C135—C134—H134	121.00
C13—C14—C15	119.5 (3)	C133—C134—H134	121.00
Ru1—C14—C15	71.09 (19)	C136—C135—H135	120.00
Ru1—C14—C13	71.26 (18)	C134—C135—H135	120.00
Ru1—C15—C16	72.22 (18)	C131—C136—H136	119.00
Ru1—C15—C14	71.81 (19)	C135—C136—H136	118.00
C14—C15—C16	120.7 (3)	C141—C142—H142	119.00
Ru1—C16—C15	70.44 (18)	C143—C142—H142	118.00
Ru1—C16—C11	73.51 (17)	C142—C143—H143	120.00
C11—C16—C15	120.0 (3)	C144—C143—H143	120.00
O1—C17—N1	127.2 (3)	C145—C144—H144	121.00
O2—C17—N1	107.5 (3)	C143—C144—H144	120.00
O1—C17—O2	125.4 (4)	C144—C145—H145	120.00
C19—C18—C20	112.6 (4)	C146—C145—H145	120.00
O2—C18—C19	102.1 (3)	C141—C146—H146	119.00
O2—C18—C20	115.1 (3)	C145—C146—H146	118.00
C1—C6—H6A	110.00	C21—C22—H22C	111.00
C1—C6—H6B	109.00	C21—C22—H22A	111.00
C1—C6—H6C	109.00	C21—C22—H22B	111.00
H6A—C6—H6B	110.00	H22A—C22—H22C	108.00
H6A—C6—H6C	110.00	H22B—C22—H22C	108.00
H6B—C6—H6C	109.00	H22A—C22—H22B	108.00
C2—C7—H7A	109.00	C21—C23—H23A	110.00
C2—C7—H7B	109.00	C21—C23—H23C	110.00
C2—C7—H7C	110.00	H23A—C23—H23B	108.00
H7A—C7—H7B	109.00	C21—C23—H23B	110.00
H7A—C7—H7C	110.00	H23B—C23—H23C	108.00
H7B—C7—H7C	110.00	H23A—C23—H23C	109.00
C3—C8—H8A	110.00	C111—B1—C131	107.8 (2)
C3—C8—H8B	109.00	C111—B1—C141	111.0 (2)
C3—C8—H8C	110.00	C121—B1—C141	109.1 (2)
H8A—C8—H8B	109.00	C131—B1—C141	106.2 (2)
H8A—C8—H8C	110.00	C121—B1—C131	113.5 (2)
H8B—C8—H8C	109.00	C111—B1—C121	109.2 (2)
C4—C9—H9A	109.00		
			
C2—Ru1—C1—C5	−117.7 (2)	C2—Ru1—C14—C13	−51.8 (4)
C2—Ru1—C1—C6	121.4 (4)	C2—Ru1—C14—C15	176.5 (3)
C3—Ru1—C1—C2	37.46 (18)	C3—Ru1—C14—C13	−74.6 (2)
C3—Ru1—C1—C5	−80.22 (19)	C3—Ru1—C14—C15	153.64 (19)
C3—Ru1—C1—C6	158.8 (3)	C4—Ru1—C14—C13	−113.39 (19)
C4—Ru1—C1—C2	80.19 (19)	C4—Ru1—C14—C15	114.9 (2)
C4—Ru1—C1—C5	−37.50 (17)	C5—Ru1—C14—C13	−152.41 (18)
C4—Ru1—C1—C6	−158.5 (3)	C5—Ru1—C14—C15	75.8 (2)
C5—Ru1—C1—C2	117.7 (2)	C11—Ru1—C14—C13	65.85 (19)
C5—Ru1—C1—C6	−121.0 (4)	C11—Ru1—C14—C15	−65.9 (2)
C11—Ru1—C1—C2	−96.97 (18)	C12—Ru1—C14—C13	29.30 (18)
C11—Ru1—C1—C5	145.35 (17)	C12—Ru1—C14—C15	−102.5 (2)
C11—Ru1—C1—C6	24.4 (3)	C13—Ru1—C14—C15	−131.8 (3)
C12—Ru1—C1—C2	−61.5 (2)	C15—Ru1—C14—C13	131.8 (3)
C12—Ru1—C1—C5	−179.17 (16)	C16—Ru1—C14—C13	102.5 (2)
C12—Ru1—C1—C6	59.9 (3)	C16—Ru1—C14—C15	−29.26 (19)
C14—Ru1—C1—C2	150.4 (3)	C1—Ru1—C15—C14	−158.21 (19)
C14—Ru1—C1—C5	32.7 (4)	C1—Ru1—C15—C16	69.7 (2)
C14—Ru1—C1—C6	−88.2 (4)	C3—Ru1—C15—C14	−53.1 (3)
C15—Ru1—C1—C2	−172.76 (18)	C3—Ru1—C15—C16	174.9 (2)
C15—Ru1—C1—C5	69.6 (2)	C4—Ru1—C15—C14	−80.3 (2)
C15—Ru1—C1—C6	−51.4 (3)	C4—Ru1—C15—C16	147.63 (17)
C16—Ru1—C1—C2	−135.98 (18)	C5—Ru1—C15—C14	−120.6 (2)
C16—Ru1—C1—C5	106.34 (18)	C5—Ru1—C15—C16	107.31 (19)
C16—Ru1—C1—C6	−14.6 (3)	C11—Ru1—C15—C14	102.7 (2)
C1—Ru1—C2—C3	118.1 (3)	C11—Ru1—C15—C16	−29.40 (17)
C1—Ru1—C2—C7	−120.9 (4)	C12—Ru1—C15—C14	66.2 (2)
C3—Ru1—C2—C1	−118.1 (3)	C12—Ru1—C15—C16	−65.82 (19)
C3—Ru1—C2—C7	121.0 (4)	C13—Ru1—C15—C14	29.31 (19)
C4—Ru1—C2—C1	−80.65 (19)	C13—Ru1—C15—C16	−102.8 (2)
C4—Ru1—C2—C3	37.48 (17)	C14—Ru1—C15—C16	−132.1 (3)
C4—Ru1—C2—C7	158.5 (4)	C16—Ru1—C15—C14	132.1 (3)
C5—Ru1—C2—C1	−37.68 (18)	C1—Ru1—C16—C11	100.78 (19)
C5—Ru1—C2—C3	80.44 (19)	C1—Ru1—C16—C15	−128.0 (2)
C5—Ru1—C2—C7	−158.5 (4)	C2—Ru1—C16—C11	65.5 (2)
C11—Ru1—C2—C1	98.05 (19)	C2—Ru1—C16—C15	−163.3 (2)
C11—Ru1—C2—C3	−143.82 (18)	C4—Ru1—C16—C11	171.78 (19)
C11—Ru1—C2—C7	−22.8 (4)	C4—Ru1—C16—C15	−57.0 (3)
C12—Ru1—C2—C1	137.00 (18)	C5—Ru1—C16—C11	141.57 (18)
C12—Ru1—C2—C3	−104.87 (19)	C5—Ru1—C16—C15	−87.2 (2)
C12—Ru1—C2—C7	16.2 (4)	C11—Ru1—C16—C15	131.2 (3)
C13—Ru1—C2—C1	173.55 (18)	C12—Ru1—C16—C11	−28.49 (17)
C13—Ru1—C2—C3	−68.3 (2)	C12—Ru1—C16—C15	102.7 (2)
C13—Ru1—C2—C7	52.7 (4)	C13—Ru1—C16—C11	−65.26 (19)
C14—Ru1—C2—C1	−150.0 (3)	C13—Ru1—C16—C15	66.0 (2)
C14—Ru1—C2—C3	−31.9 (4)	C14—Ru1—C16—C11	−102.1 (2)
C14—Ru1—C2—C7	89.2 (4)	C14—Ru1—C16—C15	29.1 (2)
C16—Ru1—C2—C1	62.5 (2)	C15—Ru1—C16—C11	−131.2 (3)
C16—Ru1—C2—C3	−179.38 (17)	C18—O2—C17—O1	5.9 (5)
C16—Ru1—C2—C7	−58.4 (4)	C18—O2—C17—N1	−173.9 (3)
C1—Ru1—C3—C2	−37.31 (19)	C17—O2—C18—C19	160.2 (3)
C1—Ru1—C3—C4	80.62 (18)	C17—O2—C18—C20	−77.6 (4)
C1—Ru1—C3—C8	−159.0 (4)	C17—N1—C11—Ru1	−81.3 (4)
C2—Ru1—C3—C4	117.9 (3)	C17—N1—C11—C16	9.3 (5)
C2—Ru1—C3—C8	−121.7 (4)	C11—N1—C17—O1	0.2 (5)
C4—Ru1—C3—C2	−117.9 (3)	C17—N1—C11—C12	−168.2 (3)
C4—Ru1—C3—C8	120.4 (4)	C11—N1—C17—O2	179.9 (3)
C5—Ru1—C3—C2	−80.1 (2)	C5—C1—C2—C3	0.3 (3)
C5—Ru1—C3—C4	37.81 (17)	C6—C1—C2—Ru1	−120.5 (3)
C5—Ru1—C3—C8	158.2 (4)	C6—C1—C2—C3	178.3 (3)
C11—Ru1—C3—C2	55.8 (3)	C5—C1—C2—C7	−178.6 (3)
C11—Ru1—C3—C4	173.76 (17)	Ru1—C1—C5—C4	60.9 (2)
C11—Ru1—C3—C8	−65.8 (4)	Ru1—C1—C5—C10	−122.7 (3)
C12—Ru1—C3—C2	90.2 (2)	C2—C1—C5—Ru1	−61.43 (19)
C12—Ru1—C3—C4	−151.87 (17)	C2—C1—C5—C4	−0.5 (3)
C12—Ru1—C3—C8	−31.5 (4)	C2—C1—C5—C10	175.9 (3)
C13—Ru1—C3—C2	129.3 (2)	C6—C1—C5—Ru1	120.6 (3)
C13—Ru1—C3—C4	−112.80 (18)	C6—C1—C2—C7	−0.6 (5)
C13—Ru1—C3—C8	7.6 (4)	Ru1—C1—C2—C3	−61.2 (2)
C14—Ru1—C3—C2	166.7 (2)	Ru1—C1—C2—C7	119.9 (3)
C14—Ru1—C3—C4	−75.4 (2)	C5—C1—C2—Ru1	61.5 (2)
C14—Ru1—C3—C8	45.0 (4)	C6—C1—C5—C4	−178.5 (3)
C15—Ru1—C3—C2	−158.5 (3)	C6—C1—C5—C10	−2.1 (5)
C15—Ru1—C3—C4	−40.6 (3)	C1—C2—C3—C4	0.0 (3)
C15—Ru1—C3—C8	79.8 (4)	C7—C2—C3—C4	178.9 (3)
C1—Ru1—C4—C3	−80.17 (19)	C1—C2—C3—C8	−177.5 (3)
C1—Ru1—C4—C5	37.38 (17)	C7—C2—C3—Ru1	−119.7 (3)
C1—Ru1—C4—C9	159.2 (3)	Ru1—C2—C3—C4	−61.4 (2)
C2—Ru1—C4—C3	−37.54 (19)	Ru1—C2—C3—C8	121.1 (3)
C2—Ru1—C4—C5	80.0 (2)	C1—C2—C3—Ru1	61.4 (2)
C2—Ru1—C4—C9	−158.2 (3)	C7—C2—C3—C8	1.4 (5)
C3—Ru1—C4—C5	117.6 (2)	C2—C3—C4—C9	−177.8 (3)
C3—Ru1—C4—C9	−120.7 (3)	C8—C3—C4—Ru1	−121.0 (3)
C5—Ru1—C4—C3	−117.6 (2)	Ru1—C3—C4—C5	−61.8 (2)
C5—Ru1—C4—C9	121.8 (3)	Ru1—C3—C4—C9	120.8 (3)
C12—Ru1—C4—C3	48.1 (3)	C2—C3—C4—Ru1	61.4 (2)
C12—Ru1—C4—C5	165.61 (19)	C2—C3—C4—C5	−0.3 (3)
C12—Ru1—C4—C9	−72.6 (3)	C8—C3—C4—C5	177.2 (3)
C13—Ru1—C4—C3	82.9 (2)	C8—C3—C4—C9	−0.2 (5)
C13—Ru1—C4—C5	−159.61 (17)	C3—C4—C5—Ru1	61.6 (2)
C13—Ru1—C4—C9	−37.8 (3)	C9—C4—C5—Ru1	−121.0 (3)
C14—Ru1—C4—C3	121.36 (19)	C3—C4—C5—C1	0.5 (3)
C14—Ru1—C4—C5	−121.09 (19)	C3—C4—C5—C10	−175.9 (3)
C14—Ru1—C4—C9	0.7 (3)	Ru1—C4—C5—C1	−61.1 (2)
C15—Ru1—C4—C3	159.73 (19)	Ru1—C4—C5—C10	122.5 (3)
C15—Ru1—C4—C5	−82.7 (2)	C9—C4—C5—C1	177.9 (3)
C15—Ru1—C4—C9	39.0 (3)	C9—C4—C5—C10	1.5 (5)
C16—Ru1—C4—C3	−165.1 (2)	C16—C11—C12—C13	4.1 (4)
C16—Ru1—C4—C5	−47.5 (3)	N1—C11—C12—C13	−178.3 (3)
C16—Ru1—C4—C9	74.3 (3)	C16—C11—C12—Ru1	−51.3 (2)
C1—Ru1—C5—C4	−118.1 (2)	Ru1—C11—C12—C13	55.4 (2)
C1—Ru1—C5—C10	121.0 (4)	N1—C11—C12—Ru1	126.3 (3)
C2—Ru1—C5—C1	37.56 (17)	C12—C11—C16—C15	−3.8 (4)
C2—Ru1—C5—C4	−80.54 (19)	Ru1—C11—C16—C15	−55.0 (2)
C2—Ru1—C5—C10	158.5 (3)	N1—C11—C16—Ru1	−126.4 (3)
C3—Ru1—C5—C1	80.44 (19)	N1—C11—C16—C15	178.7 (3)
C3—Ru1—C5—C4	−37.66 (17)	C12—C11—C16—Ru1	51.1 (2)
C3—Ru1—C5—C10	−158.6 (3)	Ru1—C12—C13—C14	54.9 (2)
C4—Ru1—C5—C1	118.1 (2)	C11—C12—C13—C14	−1.8 (4)
C4—Ru1—C5—C10	−120.9 (4)	C11—C12—C13—Ru1	−56.7 (2)
C11—Ru1—C5—C1	−54.3 (2)	C12—C13—C14—Ru1	−55.0 (2)
C11—Ru1—C5—C4	−172.40 (17)	Ru1—C13—C14—C15	54.2 (2)
C11—Ru1—C5—C10	66.7 (4)	C12—C13—C14—C15	−0.8 (4)
C13—Ru1—C5—C1	158.3 (2)	C13—C14—C15—Ru1	−54.3 (2)
C13—Ru1—C5—C4	40.2 (3)	Ru1—C14—C15—C16	55.3 (3)
C13—Ru1—C5—C10	−80.8 (4)	C13—C14—C15—C16	1.1 (4)
C14—Ru1—C5—C1	−166.20 (18)	C14—C15—C16—C11	1.3 (4)
C14—Ru1—C5—C4	75.7 (2)	Ru1—C15—C16—C11	56.4 (2)
C14—Ru1—C5—C10	−45.2 (3)	C14—C15—C16—Ru1	−55.1 (3)
C15—Ru1—C5—C1	−128.39 (18)	C116—C111—C112—H112	−178.00
C15—Ru1—C5—C4	113.51 (18)	B1—C111—C112—H112	5.00
C15—Ru1—C5—C10	−7.4 (3)	C112—C111—C116—H116	178.00
C16—Ru1—C5—C1	−88.98 (19)	B1—C111—C116—H116	−5.00
C16—Ru1—C5—C4	152.92 (17)	C111—C112—C113—H113	179.00
C16—Ru1—C5—C10	32.0 (3)	H112—C112—C113—C114	179.00
C1—Ru1—C11—N1	23.4 (3)	H112—C112—C113—H113	−1.00
C1—Ru1—C11—C12	134.33 (18)	C112—C113—C114—H114	180.00
C1—Ru1—C11—C16	−92.70 (19)	H113—C113—C114—C115	−180.00
C2—Ru1—C11—N1	−17.6 (3)	H113—C113—C114—H114	1.00
C2—Ru1—C11—C12	93.3 (2)	C113—C114—C115—H115	−179.00
C2—Ru1—C11—C16	−133.7 (2)	H114—C114—C115—C116	−180.00
C3—Ru1—C11—N1	−50.8 (4)	H114—C114—C115—H115	0.00
C3—Ru1—C11—C12	60.1 (2)	C114—C115—C116—H116	−179.00
C3—Ru1—C11—C16	−166.97 (19)	H115—C115—C116—C111	−180.00
C5—Ru1—C11—N1	56.1 (3)	H115—C115—C116—H116	1.00
C5—Ru1—C11—C12	166.98 (18)	C126—C121—C122—H122	−178.00
C5—Ru1—C11—C16	−60.1 (2)	B1—C121—C122—H122	0.00
C12—Ru1—C11—N1	−110.9 (3)	C122—C121—C126—H126	178.00
C12—Ru1—C11—C16	133.0 (3)	B1—C121—C126—H126	1.00
C13—Ru1—C11—N1	−140.3 (3)	C121—C122—C123—H123	179.00
C13—Ru1—C11—C12	−29.39 (17)	H122—C122—C123—C124	178.00
C13—Ru1—C11—C16	103.6 (2)	H122—C122—C123—H123	−1.00
C14—Ru1—C11—N1	−177.2 (3)	C122—C123—C124—H124	179.00
C14—Ru1—C11—C12	−66.26 (19)	H123—C123—C124—C125	180.00
C14—Ru1—C11—C16	66.7 (2)	H123—C123—C124—H124	−1.00
C15—Ru1—C11—N1	146.0 (3)	C123—C124—C125—H125	−179.00
C15—Ru1—C11—C12	−103.2 (2)	H124—C124—C125—C126	−179.00
C15—Ru1—C11—C16	29.82 (19)	H124—C124—C125—H125	2.00
C16—Ru1—C11—N1	116.2 (4)	C124—C125—C126—H126	−179.00
C16—Ru1—C11—C12	−133.0 (3)	H125—C125—C126—C121	180.00
C1—Ru1—C12—C11	−65.1 (2)	H125—C125—C126—H126	−1.00
C1—Ru1—C12—C13	163.13 (17)	C136—C131—C132—H132	179.00
C2—Ru1—C12—C11	−100.03 (19)	B1—C131—C132—H132	4.00
C2—Ru1—C12—C13	128.18 (18)	C132—C131—C136—H136	−179.00
C3—Ru1—C12—C11	−140.88 (18)	B1—C131—C136—H136	−5.00
C3—Ru1—C12—C13	87.33 (19)	C131—C132—C133—H133	180.00
C4—Ru1—C12—C11	−171.06 (18)	H132—C132—C133—C134	180.00
C4—Ru1—C12—C13	57.1 (3)	H132—C132—C133—H133	0.00
C11—Ru1—C12—C13	−131.8 (3)	C132—C133—C134—H134	−180.00
C13—Ru1—C12—C11	131.8 (3)	H133—C133—C134—C135	−180.00
C14—Ru1—C12—C11	102.5 (2)	H133—C133—C134—H134	0.00
C14—Ru1—C12—C13	−29.27 (18)	C133—C134—C135—H135	180.00
C15—Ru1—C12—C11	65.62 (19)	H134—C134—C135—C136	−179.00
C15—Ru1—C12—C13	−66.18 (19)	H134—C134—C135—H135	1.00
C16—Ru1—C12—C11	28.53 (18)	C134—C135—C136—H136	180.00
C16—Ru1—C12—C13	−103.27 (19)	H135—C135—C136—C131	179.00
C2—Ru1—C13—C12	−69.5 (2)	H135—C135—C136—H136	0.00
C2—Ru1—C13—C14	158.69 (18)	C146—C141—C142—H142	179.00
C3—Ru1—C13—C12	−106.61 (18)	B1—C141—C142—H142	3.00
C3—Ru1—C13—C14	121.6 (2)	C142—C141—C146—H146	180.00
C4—Ru1—C13—C12	−146.65 (17)	B1—C141—C146—H146	−3.00
C4—Ru1—C13—C14	81.5 (2)	C141—C142—C143—H143	−180.00
C5—Ru1—C13—C12	−173.5 (2)	H142—C142—C143—C144	−179.00
C5—Ru1—C13—C14	54.7 (3)	H142—C142—C143—H143	0.00
C11—Ru1—C13—C12	29.09 (17)	C142—C143—C144—H144	−180.00
C11—Ru1—C13—C14	−102.7 (2)	H143—C143—C144—C145	180.00
C12—Ru1—C13—C14	−131.8 (3)	H143—C143—C144—H144	1.00
C14—Ru1—C13—C12	131.8 (3)	C143—C144—C145—H145	180.00
C15—Ru1—C13—C12	102.5 (2)	H144—C144—C145—C146	179.00
C15—Ru1—C13—C14	−29.31 (19)	H144—C144—C145—H145	−2.00
C16—Ru1—C13—C12	65.45 (18)	C144—C145—C146—H146	−180.00
C16—Ru1—C13—C14	−66.4 (2)	H145—C145—C146—C141	−180.00
C1—Ru1—C14—C13	−175.8 (3)	H145—C145—C146—H146	1.00
C1—Ru1—C14—C15	52.5 (4)		

Symmetry codes: (i) *x*−1, *y*, *z*; (ii) −*x*, *y*+1/2, −*z*+1/2; (iii) *x*, −*y*+1/2, *z*+1/2; (iv) −*x*+1, *y*+1/2, −*z*+1/2; (v) *x*, −*y*+1/2, *z*−1/2; (vi) *x*+1, *y*, *z*; (vii) −*x*, *y*−1/2, −*z*+1/2; (viii) *x*−1, −*y*+1/2, *z*−1/2; (ix) −*x*, −*y*, −*z*; (x) −*x*+1, *y*−1/2, −*z*+1/2; (xi) *x*+1, −*y*+1/2, *z*+1/2; (xii) −*x*, −*y*, −*z*+1.

### Hydrogen-bond geometry (Å, °)

**Table:**

*D*—H···*A*	*D*—H	H···*A*	*D*···*A*	*D*—H···*A*
N1—H1···O3	0.88	2.07	2.890 (4)	154
C6—H6C···O1	0.95	2.56	3.512 (5)	174
